# Inhibitory Effects of Sulfur Derivatives on *Leishmania tarentolae* Cell Viability and Secreted Acid Phosphatase In Vitro

**DOI:** 10.3390/microorganisms12122641

**Published:** 2024-12-19

**Authors:** Henry H. Shang, Zaryna Z. Zelaya, Christopher G. Hamaker, Marjorie A. Jones

**Affiliations:** Department of Chemistry, Illinois State University, Normal, IL 61790, USAchamake@ilstu.edu (C.G.H.)

**Keywords:** *Leishmania*, sulfonamide Schiff base derivatives, secreted acid phosphatase

## Abstract

Sulfonamide drugs were the original class of antibiotics, demonstrating the antibacterial potential of dithiocarbazate and thiosemicarbazone Schiff base derivatives of syringaldehyde and 4-hydroxy-3,5-dimethylbenzaldehyde. We synthesized unique Schiff bases via the condensation of the aldehydes with hydrazine derivatives, which allows for the easy synthesis of several related compounds. These Schiff base derivatives were tested for antileishmanial properties against the parasitic protozoan *Leishmania tarentolae*. The inhibitory properties of these sulfur compounds were tested using a series of cell viability and secreted acid phosphatase (SAP) assays. The results demonstrated that compounds ZZ1-04 and ZZ1-20 had potent inhibitory effects on parasite cell viability and SAP, an enzyme that may play a role in infectivity. These results increase our understanding of the role of sulfur in inhibiting *Leishmania*, providing more knowledge of the structural activity relationships that may prove critical for their development into possible antileishmanial treatments.

## 1. Introduction

Currently, an estimated 700,000 to 1 million new cases of leishmaniasis, the disease resulting from infection by *Leishmania*, occur worldwide annually. *Leishmania* are single-celled protozoan parasites that can infect various species, including mammals and reptiles. *Leishmania* can spread through the bites of any of the ninety sandfly vector species. The infection can manifest via organ damage, skin ulcers, or the destruction of the mucous membranes of the nose, mouth, and throat. The health consequences of leishmaniasis are disproportionately felt by the Global South and tropical climates [1]. Current treatments include miltefosine, parenteral paromomycin, and amphotericin B, but an increased frequency of treatment failure has been observed [2]. The ability of *Leishmania* to rapidly develop resistance against current antileishmanial drugs demonstrates the urgent need for new treatments.

To treat many diseases, scientists have found sulfur to be a critical element in many natural and synthetic therapeutics. The sulfonamide drugs were the first class of antibiotics, and the potent antimicrobial properties of the sulfonamide functional group (SN) indicates the antimicrobial potential of other sulfur functional groups [3,4]. Here, we synthesized some unique Schiff base compounds via the condensation of the aldehydes with the hydrazine derivatives, which allows for the easy synthesis of several related compounds. A recent report by Aytac et al. describes the synthesis and biological activity of a series of phenol-containing Schiff bases [5]. Syringaldehyde, one of the starting aldehydes used in the syntheses, has been shown to have significant biological activity [6,7]. Additionally, thiosemicarbazones and related compounds have long been known to exhibit biological activity [8,9].

**Figure 1:**
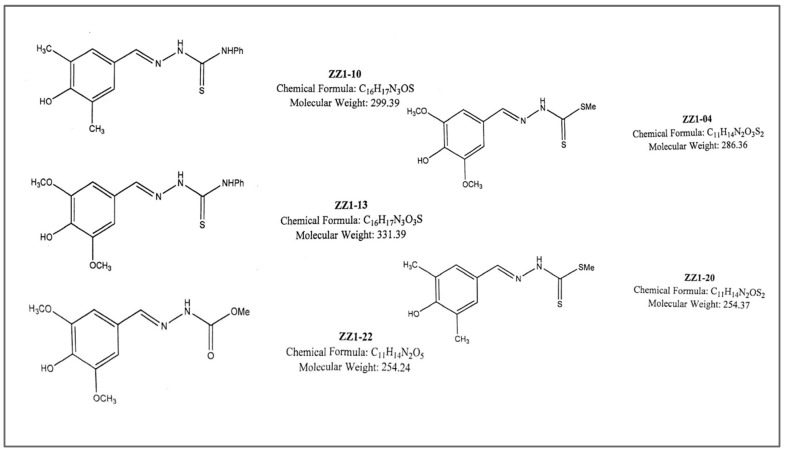
The five compounds used in this study.

Here, we now report experiments involving variations in test compound substituents and concentration so that potency against axenic *Leishmania tarentolae* in culture can be evaluated following the general protocol of Katinas et al. [10]. After growth periods, cultures were also tested for the detection of secreted acid phosphatase (SAP), an enzyme reported to play a role in promoting *Leishmania* infectivity [11]. The direct effect of compounds (Figure 1) on the control cell-free supernatant containing the SAP activity at varying concentrations was also tested.

## 2. Materials and Methods

### 2.1. Synthesis

All reagents were reagent grade and obtained from commercial sources: Alfa Aesar (Lancashire, UK), Acros Organics (Geel, Belgium), and TCI America (Portland, OR, USA).

**Synthesis of ZZ1-04:** Methyl hydrazinecarbodithioate (10.011 mmol, 1.2234 g) and syringaldehyde (9.9978 mmol, 1.8214 g) were placed into a round bottom flask in 40 mL of ethanol. The mixture was refluxed for 2 h, cooled to room temperature, and the precipitate was collected by suction filtration and washed with diethyl ether. Yield: 2.5455 g (88.90%). ^1^H NMR (dmso-d6): δ 13.21 (s, 1H, OH); 9.03 (s, 1H, NH); 8.12 (s, 1H, CH=N); 6.99 (s, 2H, H_Ar_); 3.81 (s, 6H, OCH_3_); 2.52 ppm (s, 3H, SCH_3_).

**Synthesis of ZZ1-10.** In a round bottom flask, 4-phenyl-3-thiosemicarbazide (9.9934 mmol, 1.6712 g) and 4-hydroxy-3,5-dimethylbenzaldehyde (10.002 mmol, 1.5046 g) were added to 30 mL ethanol. The mixture was refluxed for 2.5 h, cooled to room temperature, and the precipitate was collected by suction filtration and washed with diethyl ether. Yield: 2.5313 g (84.60%). ^1^H NMR (dmso-d6): δ 11.60 (s, 1H, OH); 9.91 (s, 1H, NH); 8.71 (s, 1H, NH); 8.02 (s, 1H, CH=N); 7.57 (d, 2H, H_Ar_, J = 7.6 Hz); 7.45 (s, 2H, H_Ar_); 7.36 (t, 2H, H_Ar_, J = 7.8 Hz); 7.20 (t, 1H, H_Ar_, J = 7.4 Hz); 2.16 ppm (s, 6H, CH_3_).

**Synthesis of ZZ1-13.** A mixture of 4-phenyl-3-thiosemicarbazide (10.000 mmol, 1.6723 g) and syringaldehyde (9.9989 mmol, 1.8215 g) was placed into a round bottom flask, in 40 mL of ethanol. The mixture was refluxed for 2 h, cooled to room temperature, and the precipitate was collected by suction filtration and washed with diethyl ether. Yield: 3.0340 g (91.56%). ^1^H NMR (dmso-d6): δ 11.70 (s, 1H, OH); 9.96 (s, 1H, NH); 8.85 (s, 1H, NH); 8.04 (s, 1H, CH=N); 7.57 (d, 2H, H_Ar_, J = 8.4 Hz); 7.37 (t, 2H, H_Ar_, J = 7.9 Hz); 7.21 (t, 1H, H_Ar_, J = 8.2 Hz); 7.13 (s, 2H, H_Ar_); 3.82 ppm (s, 6H, OCH_3_).

**Synthesis of ZZ1-20.** In a round bottom flask, 4-hydroxy-3,5-dimethylbenzaldehyde (9.9901 mmol, 1.5004 g) and methyl hydrazinecarbodithioate (9.9983 mmol, 1.2221 g) were added to 30 mL ethanol. The mixture was refluxed for 2 h, cooled to room temperature, and the precipitate was collected by suction filtration and washed with diethyl ether. Yield: 0.6327 g (25.12%). ^1^H NMR (dmso-d6): δ 13.09 (s, 1H, OH); 8.87 (s, 1H, NH); 8.09 (s, 1H, CH=N); 7.29 (s, 2H, H_Ar_); 2.51 (s, 3H, SCH_3_); 2.19 ppm (s, 6H, CH_3_).

**Synthesis of ZZ1-22.** Methyl carbazate (9.997 mmol, 0.9005 g) and syringaldehyde (9.998 mmol, 1.8215 g) were dissolved in 30 mL of ethanol in a round bottom flask. The mixture was refluxed for 2 h, cooled to room temperature, and the precipitate was collected by suction filtration and washed with diethyl ether. Yield: 1.2846 g (50.54%). ^1^H NMR (dmso-d6): δ 10.93 (s, 1H, OH); 8.76 (s, 1H, NH); 7.89 (s, 1H, CH=N); 6.87 (s, 2H, H_Ar_); 3.78 (s, 6H, OCH_3_); 3.67 ppm (s, 3H, OCH_3_).

### 2.2. X-Ray Data Collection and Refinement

Data were collected on a Bruker APEX II CCD diffractometer at 100 (2) K using MoKα radiation (λ = 0.71073 Å). The data were processed and corrected for absorption using the Bruker SAINT V8.38A software package [12]. Structures were solved by direct methods using SHELXS-2017 and the data were refined using SHELXL-2017 [13]. All non-H atoms were refined anisotropically. Hydrogen atoms attached to carbon were assigned positions based on the geometries of their attached carbons. Hydrogen atoms bonded to oxygen and nitrogen were assigned positions based on the Fourier difference map. See Table 1 for final refinement parameters. Figures were made using ORTEP3 [14] and Mercury 4.0 [15].

### 2.3. Cell Cultures of L. tarentolae

ATCC 30143 promastigote cells were used as a test model system for our studies since Taylor et al. have determined them to be a good model [16]. The *L. tarentolae* cells were cultured in sterile brain heart infusion (BHI) medium with hemin, penicillin, and streptomycin in the dark at room temperature (~26 °C) following the methods of Morgenthaler et al. and modulated by Katinas et al. to ensure uniformity of cell quality and age [10,17]. For each experiment, the same volume of cells was inoculated into 10 mL cultures (using a stock culture). Time was considered as day 0 on the day of inoculation. All work was performed using sterile techniques.

### 2.4. MTT Cell Viability Assay

The cell viability of the *Leishmania* cultures was assessed using the 3-[4,5-dimethylthiazol-2-yl]-2,5-diphenyltetrazolium bromide (MTT) assay following the methods of Mosmann [18]. Sigma Aldrich (Burlington, MA, USA) Flatbottom 96-well plates were used for these assays, and spectrophotometric data were recorded at a wavelength of 595 nm using the Bio-Rad^®^ Microplate Reader Benchmark (Hercules, CA, USA). The A595nm values were normalized by subtracting the absorbance values of the cell-free BHI blanks and normalized to an incubation time of one hour. Values are reported as mean ± standard deviation for n = 4 replicates. Significant differences were evaluated using one-way ANOVA and considered significantly different when *p* < 0.05.

### 2.5. Dose-Dependent Effect of Test Compounds on Cell Viability

To study the dose-dependent effect of the test compound on *L. tarentolae*, cell cultures were generated by extracting 10.0 mL aliquots from the stock culture. A flask containing 10.0 mL of only fresh BHI medium (without *Leishmania*) was created for each experiment to serve as the blank. The new cell cultures were immediately treated with specific concentrations of the test compound dissolved in dimethyl sulfoxide (DMSO). All such compound solutions were freshly prepared except where indicated. The cell culture containing only DMSO (without any test compound) served as the experimental control. The final concentration of DMSO in each flask was 1% (*v*/*v*). Compound addition only occurred once per culture for all cultures. Compound addition occurred during the early-to-middle log phase of a cell culture’s growth curve.

Cell viability data for all flasks were collected using the MTT assay right before compound addition and on various days after compound addition, determined by time-dependent experiments to be when the compound started showing inhibitory effects on cell viability. Cell viability data from day 5 were used to estimate the IC_50_ of the compound.

### 2.6. SAP Enzyme Assay

Using the method of Dorsey et al. [19], 1.5 mL volumes were removed from the 7-day-old culture flasks. The cells were centrifuged for thirty seconds at 10,000× *g* using an Eppendorf tabletop model 5415 centrifuge (Eppendorf, Hamburg, Germany). Cell-free supernatants were removed from these samples and tested for the secreted acid phosphatase activity in 1.5 mL polypropylene tubes. BHI without any compounds or cells was used as blanks and cell-free supernatant (450 µL) was mixed with 450 µL of 0.5 M of a sodium acetate buffer (pH 4.5) for each replicate. Then, 100 µL of a freshly prepared solution of para-nitrophenyl phosphate (pNPP, 5.0 mg/mL buffer; Sigma Aldrich, (Burlington, MA, USA), as the artificial substrate for the phosphatase enzyme, was added. Replicates were incubated in the dark at room temperature (~26 °C) for 23 h. The reaction was stopped by adding 100 µL of 10 M NaOH to each sample tube. The samples were measured at A405 nm. Results, as percent of control cell supernatant, were reported as mean ± standard deviation for n = 3 replicates. Significant differences were evaluated using one-way ANOVA or Students *t*-test (when only two groups were being compared considered significantly different when *p* < 0.05.

### 2.7. Dose-Dependent Direct Effect on SAP Activity

To study the direct effect of the test compound on SAP enzyme activity, 1.5 mL volumes were removed from a 7-day-old control cell culture flask without the presence of compounds or dimethyl sulfoxide (DMSO). The cells were centrifuged for thirty seconds at 10,000× *g* using an Eppendorf tabletop model 5415 centrifuge. Cell-free supernatants were removed from these samples. Experimental groups were treated with addition of specific concentrations of the test compound dissolved in DMSO. All such compound solutions were freshly prepared unless otherwise indicated. The final concentration of DMSO in each replicate was 1%. BHI without any compounds or cells was used as blanks. Cell-free supernatant without any compounds added retroactively was used as the experimental control.

### 2.8. Comparing Secreted and Intracellular Leishmania Acid Phosphatase Activity

To determine the effect of a single addition of the test compound on extracellular relative to intracellular acid phosphatase activity, 1.5 mL volumes were removed from 7-day-old culture flasks. The cultures were prepared according to the methods of Morgenthaler et al. [17], where the cell cultures were treated with specific concentrations of the freshly prepared test compound dissolved in dimethyl sulfoxide (DMSO). The cell culture containing only DMSO (without any test compound) served as the experimental control. The final concentration of DMSO in each flask was 1%. The cells were centrifuged for thirty seconds at 10,000× *g* using an Eppendorf tabletop model 5415 centrifuge. Cell-free supernatants were removed from these samples, and the 450 µL of cell-free supernatant was mixed with 450 µL of 0.5 M of a sodium acetate buffer (pH 4.5) for each replicate (n = 3). BHI without any compounds or cells was used as blanks. Each cell pellet was then resuspended in 900 µL of buffer. Resuspended cell pellets with 100 µL of 10 M NaOH added before the addition of substrate were used as blanks. Then, 100 µL of freshly prepared solution of para-nitrophenyl phosphate (pNPP, 5.0 mg/mL buffer; Sigma Aldrich) were added. Replicates were incubated in the dark at room temperature (~26 °C) for 23 h. The assay was stopped by adding 100 µL of 10 M NaOH to each sample tube. After incubation, the resuspended cell pellets were centrifuged for thirty seconds at 10,000× *g* using an Eppendorf tabletop model 5415 centrifuge and the supernatant was removed from each sample prior to spectroscopy.

All samples were measured at A405 nm. Results are reported as mean ± standard deviation for n = 3 replicates. The cell-free supernatant replicates measured the secreted (extracellular) acid phosphatase activity, while the resuspended pellet replicates measured the intracellular acid phosphatase activity. By comparing the % of control for each experimental group (cell-free supernatant versus resuspended pellet), the effect of the test compound on secreted and intracellular acid phosphatase activities can be compared.

## 3. Results and Discussion

### 3.1. Synthesis and Characterization of Compounds

All five compounds were synthesized by refluxing the appropriate aldehyde and hydrazine derivative in ethanol for approximately two hours, cooling the mixture to room temperature, and isolating the resulting precipitate via vacuum filtration. Yields for the compounds ranged from about 25% to greater than 90%. All of the compounds showed expected resonances in the ^1^H NMR. Compounds ZZ1-04 and ZZ1-13 were characterized by single-crystal X-ray crystallography. These two compounds were the only ones that yielded crystals of sufficient quality for crystallographic analysis.

### 3.2. Crystallographic Analysis of ZZ1-04 and ZZ1-13

**Figure 2:**
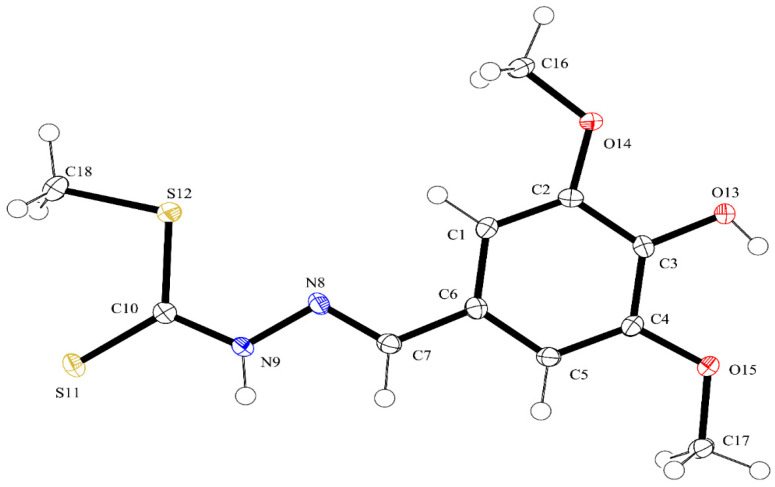
ORTEP diagram of ZZ1-04. Thermal ellipsoids shown at the 50% probability level and hydrogen atoms as spheres of arbitrary size.

**Figure 3:**
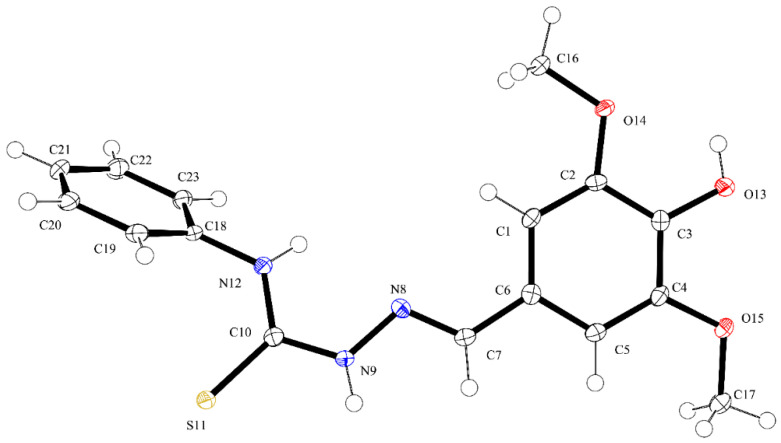
ORTEP diagram of ZZ1-13. Thermal ellipsoids shown at the 50% probability level and hydrogen atoms as spheres of arbitrary size.

**Figure 4:**
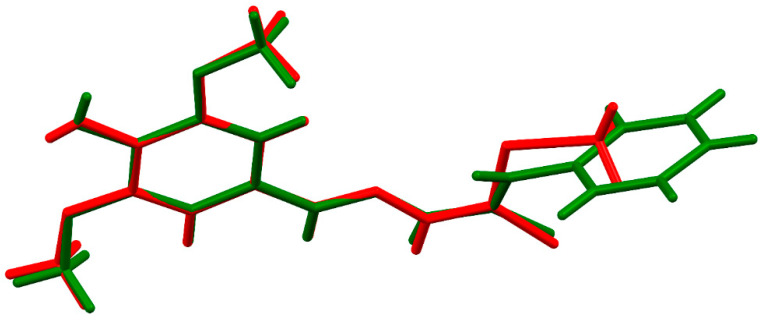
Capped stick overlays of ZZ1-04 (green) and ZZ1-13 (red) showing the similarities in the structures.

ORTEP diagrams of ZZ1-04 and ZZ1-13 are shown in Figure 2 and Figure 3, respectively. Both molecules crystallized in the monoclinic space group *P*2_1_/*c* with four molecules per unit cell. All of the bond lengths and angles (Table 2 and Table 3) are similar to other Schiff base complexes previously analyzed [20,21]. Both complexes have remarkably similar structures (Figure 4), with the only significant differences being in the group attached to the thiocarbonyl, methylthio in ZZ1-04, and aminophenyl in ZZ1-13. The C10–S12/N12 distances are quite different, as would be expected, with ZZ1-04 having a C10–S12 distance of 1.7475 (13) Å and ZZ1-13 having a C10–N12 distance of 1.351 (2) Å. The differing substituents also give rise to different C10–S12/N12–C18 angles; 101.38 (6)° in ZZ1-04 and 125.16 (14) in ZZ1-13. These differences as well as the significant similarities in the molecules can be seen in Figure 4.

**Figure 5:**
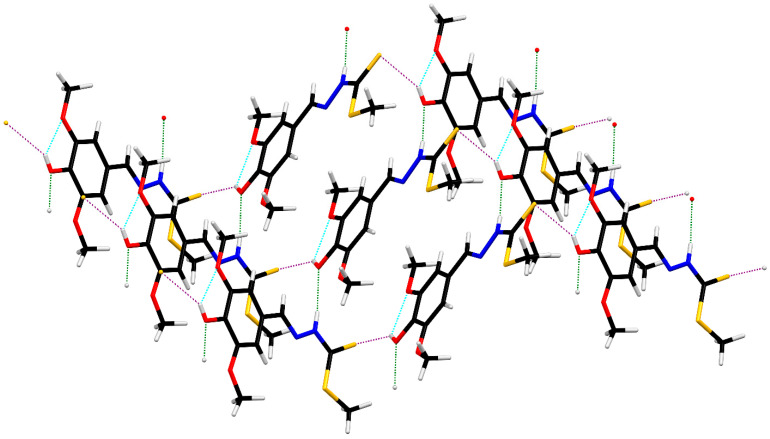
Hydrogen-bonded zigzag sheets in ZZ1-04.

However, the two molecules have different three-dimensional hydrogen-bonding patterns in the crystalline state. In ZZ1-04, the hydroxyl group forms an intermolecular hydrogen bond with the thiocarbonyl sulfur of a neighboring molecule while the thioamide N–H forms an intermolecular hydrogen bond with the hydroxy oxygen of a neighboring molecule (Table 4). These interactions result in the formation of zigzag sheets in the crystalline lattice (Figure 5).

**Figure 6:**
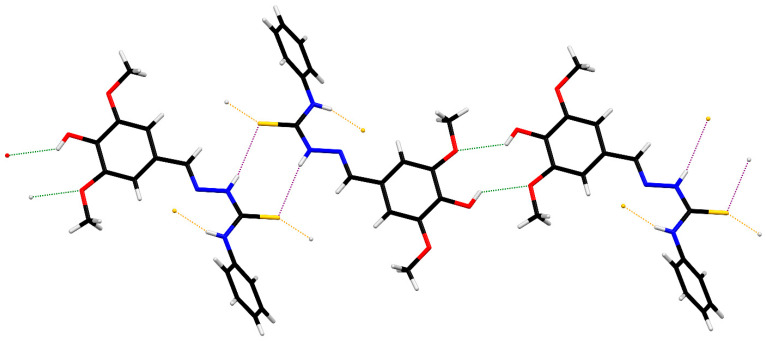
Hydrogen-bonded chains in ZZ1-13.

**Figure 7:**
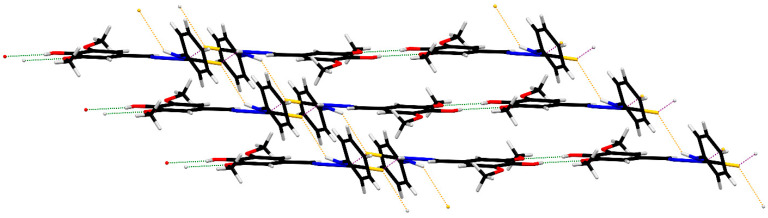
Hydrogen-bonded layers in ZZ1-13.

In ZZ1-13, there are $R_{2}^{2}(8)$ thioamide dimers (purple) as well as $R_{2}^{2}(10)$ dimers (green) formed with the hydroxyl group and *ortho*-methoxy groups of neighboring molecules (Table 5, Figure 6) which form two-dimensional ribbons. The ribbons are connected into larger layers via N–H^…^S hydrogen bonds (orange), as seen in Figure 7.

### 3.3. Growth Curves Following Addition of ZZ1-10 and ZZ1-13

**Figure 8:**
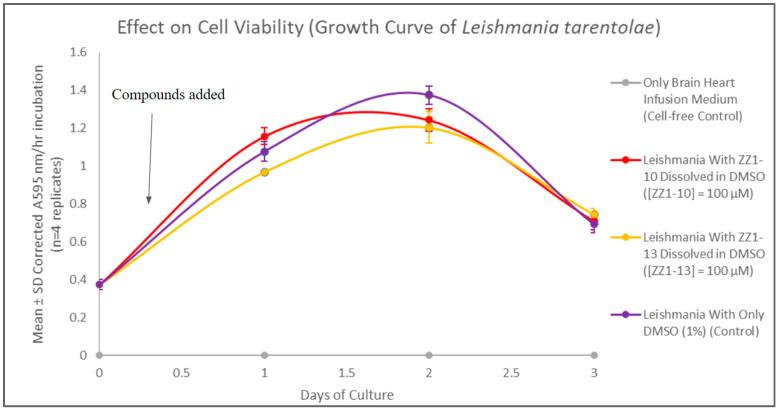
Results of the MTT viability assays over the course of the growth cycle. Corrected mean and SD values (n = 4) are given for the 3-day growth period. The final concentration of compounds ZZ1-10 and ZZ1-13 in their respective flasks are both 100 µM.

The MTT spectrophotometric assay, utilizing 3-(4,5-dimethylthiazol-2-yl)-2,5-diphenyltetrazolium bromide (MTT reagent), was used to evaluate cell viability and generate growth curves for control (DMSO solvent only) and experimental cultures treated with ZZ1-10 or with ZZ1-13 dissolved in DMSO. There were no statistically significant differences between the growth curves for the control cells and these two experimental cultures. These results (Figure 8) show that the presence of methyl versus methoxy groups on the benzene ring, by themselves, do not have significant effects on cell viability.

### 3.4. ZZ1-22 MTT and SAP Assays

**Figure 9:**
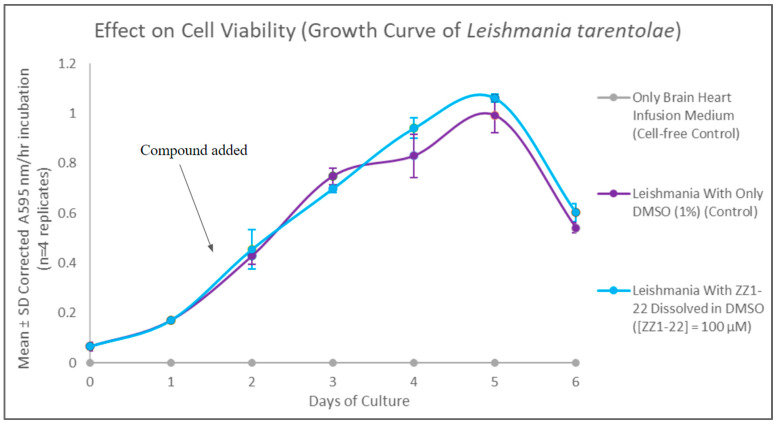
Results of the MTT viability assays over the course of the growth cycle. Corrected mean and SD values (n = 4) are given for the 6-day growth period. The final concentration of compound ZZ1-22 in the flask is 100 µM.

The MTT assay was used to evaluate cell viability and generate growth curves for control and experimental cultures treated with ZZ1-22 (Figure 9). There were no statistically significant differences between the growth curves for the control and experimental group. These results show that the presence of a carbonyl group does not have significant effects on cell viability.

**Figure 10:**
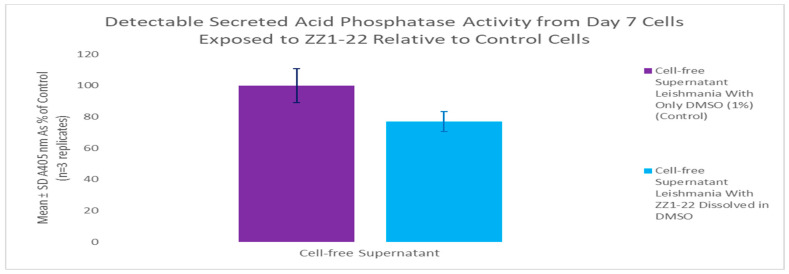
Secreted acid phosphatase (SAP) assay following growth cycle study (cell culture age: 7 days). Values are the mean and SD values as % of control culture for n = 3 replicates.

A secreted acid phosphatase (SAP) assay was performed using cell-free supernatant from day 7 of the growth curve. ZZ1-22 had a statistically significant inhibitory effect of 23% reduction in detectable SAP enzyme activity relative to control cell SAP activity (Figure 10). These results show that the presence of a carbonyl group does impact detectable SAP activity.

### 3.5. ZZ1-04 and Assays

#### 3.5.1. ZZ1-04 and Cell Viability

**Figure 11:**
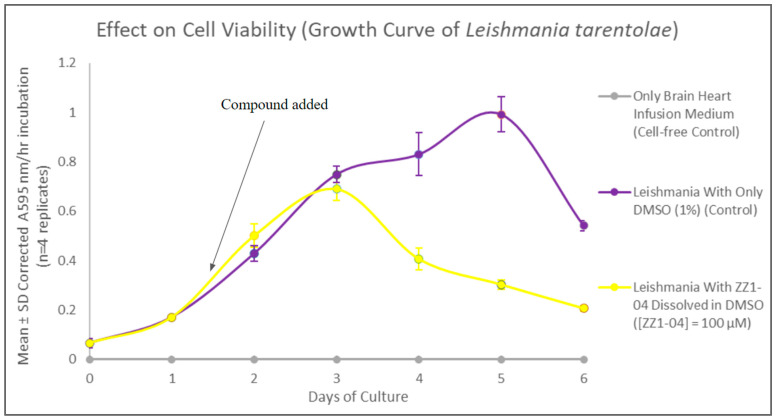
Corrected mean and SD values (n = 4) for the MTT viability assays are shown for the 6-day growth period. The final concentration of compound ZZ1-04 in the flask is 100 µM.

The MTT assay was used to evaluate cell viability and generate growth curves for control and experimental cultures treated with ZZ1-04, as shown in Figure 11. At maximum inhibitory effect (day 5), the ZZ1-04 addition resulted in a statistically significant 69.5% decrease in *Leishmania* cell viability. Comparing these results to those of the three previous compounds indicate that the presence of a thioester group does have significant effects on cell viability.

#### 3.5.2. ZZ1-04 and Secreted Acid Phosphatase Activity

**Figure 12:**
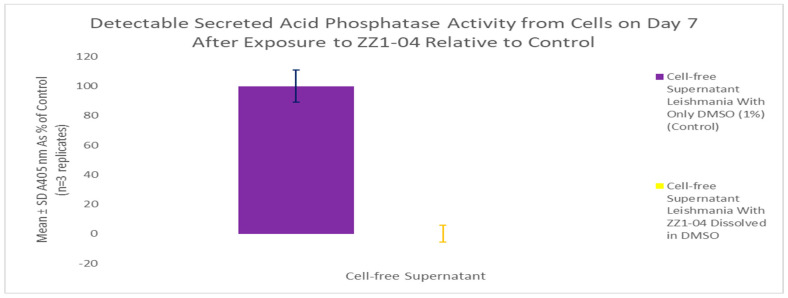
SAP assay following growth curve study ± 100 µM ZZ1-04 (cell culture age: 7 days). Values are the mean and SD for n = 3 replicates.

A secreted acid phosphatase (SAP) assay was performed using the day 7 culture as shown in Figure 12. ZZ1-04 had a statistically significant 100% inhibitory effect on detectable SAP enzyme activity. These results indicate that the presence of a methylthio group greatly impacts detectable SAP activity.

**Figure 13:**
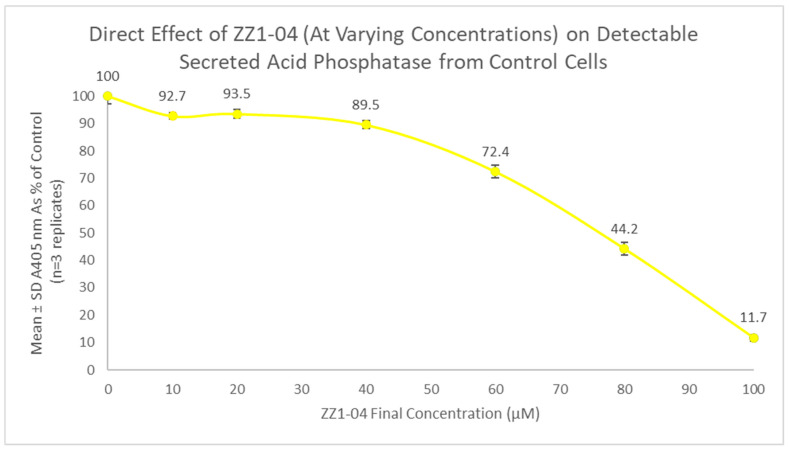
Direct dose-dependent effect of ZZ1-04 on detectable SAP activity (cell culture age: 7 days). Values are the mean and SD for n = 3 replicates.

Following this initial study, it was hypothesized that compound ZZ1-04 could have one or more of the following probable effects on detectable SAP activity: decrease the ability of the cells to make the enzyme, decrease the ability of the cells to secrete the enzyme, and/or directly inhibit the secreted enzyme. Thus, we conducted an experiment to test for direct inhibition of the secreted enzyme using cells not exposed to the test compound. Direct effect dose-dependent experiments for the SAP assay were conducted, and the values are shown in Figure 13. The estimated IC_50_ of the direct effect of ZZ1-04 on detectable SAP enzyme activity was between 60 and 80 µM. All tested direct effect concentrations had a statistically significant effect, with the 10 μM having a direct inhibitory effect of 7.3%. ZZ1-04 at 100 μM had a statistically significant direct inhibitory effect on control *Leishmania* cell-free supernatant SAP of 88.3%.

**Figure 14:**
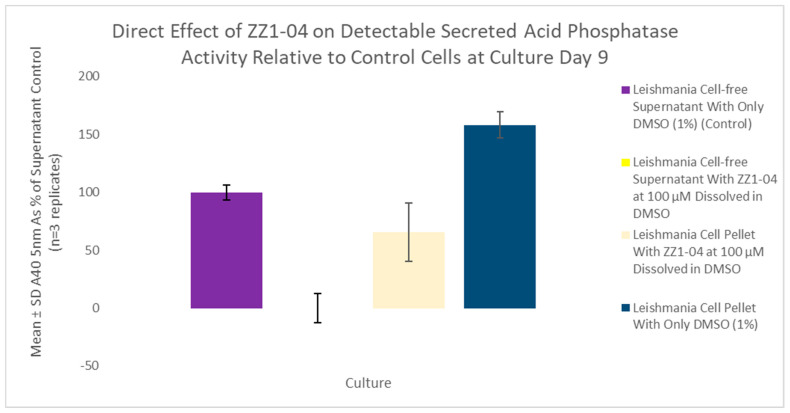
SAP assay comparing extracellular and intracellular acid phosphatase activity (cell culture age: 9 days). Values are the mean and standard deviation for n = 3 replicates.

It was hypothesized that compound ZZ1-04 could decrease the ability of the cells to secrete the acid phosphatase enzyme due to the disparity in results between the indirect and direct effect studies. Thus, SAP assays that compared the extracellular (secreted) and intracellular acid phosphatases were conducted, and the values are shown in Figure 14. ZZ1-04 had a statistically significant reduction on the detectable extracellular active acid phosphatase activity by 100% and a statistically significant reduction on the detectable intracellular active acid phosphatase activity by 58.5%. These results suggest that ZZ1-04 inhibits both the production of the SAP enzyme and its direct extracellular activity.

### 3.6. ZZ1-20 MTT and SAP Assays

**Figure 15:**
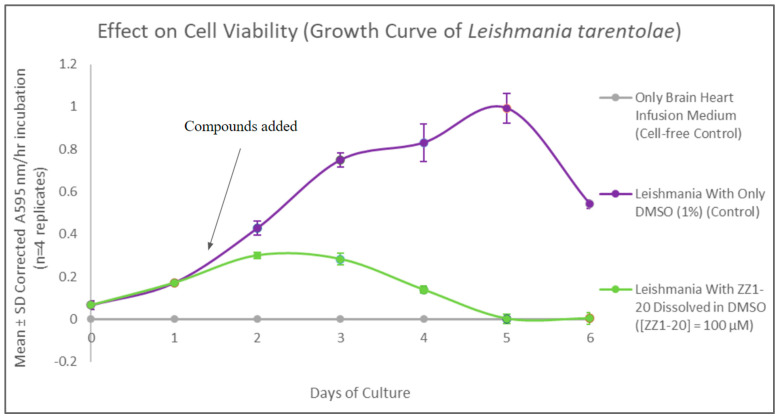
Results of the MTT viability assays over the course of the growth cycle. Corrected mean and SD values (n = 4) are given for the 6-day growth period. The final concentration of compound ZZ1-20 in the flask is 100 µM.

The MTT assay was used to evaluate cell viability and generate growth curves for control and experimental cultures treated with ZZ1-20. At maximum inhibitory effect (day 5), ZZ1-20 had a statistically significant 99.8% decrease in *Leishmania* cell viability. Comparing these results to those of the previous compounds shows that the presence of a thioester group and the ring methyl groups does result in significant inhibitory effects on cell viability, as shown in Figure 15.

**Figure 16:**
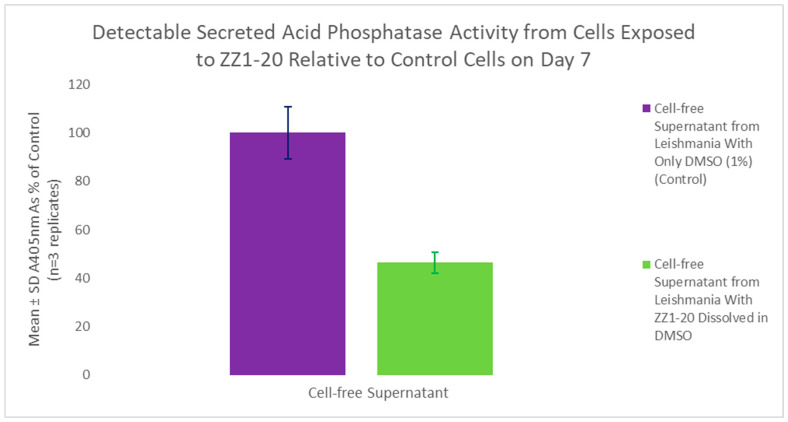
SAP assay following growth cycle study (cell culture age: 7 days) using ZZ1-20. Values are the mean and SD for n = 3 replicates.

A secreted acid phosphatase (SAP) assay was performed using the flask from the initial growth curve. ZZ1-20 had a statistically significant 53.6% inhibitory effect on detectable SAP enzyme activity, as shown in Figure 16.

**Figure 17:**
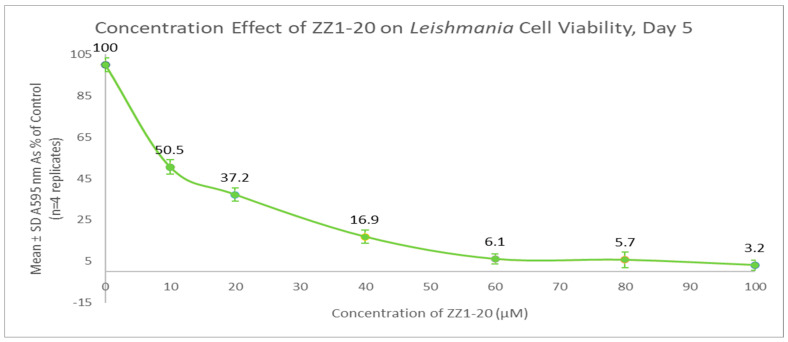
Dose-dependent effect of ZZ1-20 on cell viability (on day 5 cultures). Values are the mean and SD for n = 4 replicates.

**Figure 18:**
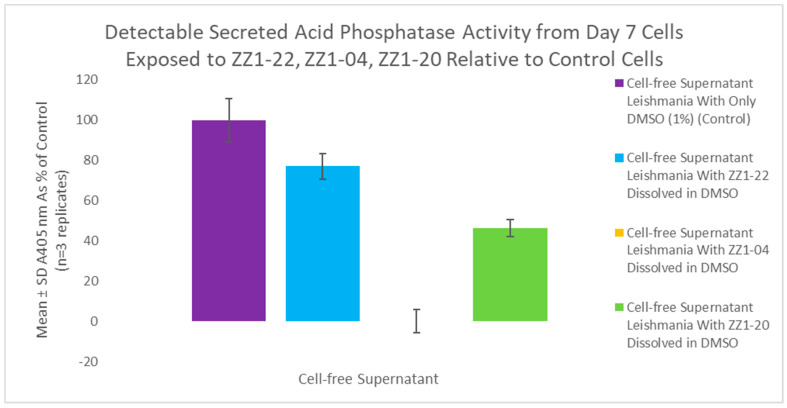
Comparative SAP assay following growth cycle study (cell culture age: 7 days) using ZZ1-22, ZZ1-04, or ZZ1-20 relative to control with DMSO only. Values are the mean and SD for n = 3 replicates.

Growth curves from dose-dependent experiments for ZZ1-20 are shown in Figure 17. The IC_50_ value for ZZ1-20 was ≅ 10 µM for day 5 cultures. All values are statistically significant from the control. A comparison of the ZZ1-22, ZZ1-04, and ZZ1-20 SAP assay data are shown in Figure 18.

### 3.7. Stability in Solution Study for Direct Effect on SAP

The direct effect on detectable SAP activity study was conducted with compounds that had been in solution for 56 days and with freshly prepared compounds for compounds ZZ1-22, ZZ1-04, and ZZ1-20. Freshly prepared and old ZZ1-04 had a statistically significant >90% reduction in detectable SAP activity, freshly prepared (56.2%) and old ZZ1-20 (75.9%), and old ZZ1-22 (95.3%) (Table 6).

Such a disparity in the results suggests that the ZZ1-22 compound is not stable long-term in solution, and thus should always be prepared fresh before usage in any experiment. Thus, the ZZ1-22 compound instability may lead to degradation products that do negatively impact cell-free SAP activity but perhaps by a different mechanism.

## 4. Conclusions

In this work, the ways different functional groups of sulfur compounds can negatively affect *Leishmania* viability in vitro have now been expanded. Our results indicate that the compounds ZZ1-04 and ZZ1-20, both of which are thiocarbazate compounds, are effective at inhibiting both cell viability and SAP activity. It appears that the presence of methoxy groups on the benzene ring is also correlated with increasing the SAP inhibitory activity by the thioester; by themselves, the methoxy groups do not demonstrate inhibitory activity on SAP. It is of interest that ZZ1-04 consistently demonstrates more potent inhibitory effects on SAP secretion and activity, which may change the potential infectivity of the *Leishmania* parasite. It is also of interest that ZZ1-20 consistently demonstrates high inhibitory potency on cell viability, which may have structural implications for antileishmanial drugs. Clearly, the mechanism of action of these Schiff base derivatives needs to be determined.

### Limitations of These Studies

All work was performed using the *L. tarentolae* species, a non-human parasite, and so we encourage those who use the human parasites to test these interesting molecules in their model systems.

## Figures and Tables

**Table 1:** Data collection parameters.

Compound	ZZ1-04	ZZ1-13
CCDC Deposit No.	2364214	2364215
Chemical formula	C_11_H_14_N_2_O_3_S_2_	C_16_H_17_N_3_O_3_S
M_r_	286.36	331.38
Crystal system, space group	Monoclinic, P2_1_/c	Monoclinic, P2_1_/c
Temperature (K)	100 (2)	100 (2)
a, b, c (Å)	5.1702 (2) 17.4274 (7) 14.5938 (6)	11.6522 (7) 5.6318 (3) 24.0789 (14)
β (°)	96.097 (2)	103.799 (4)
V (Å^3^)	1307.51 (9)	1534.52 (15)
Z	4	4
Radiation type	Mo K_α_	Mo K_α_
µ (mm^–1^)	0.409	0.230
Crystal size (mm)	0.47 × 0.18 × 0.04	0.21 × 0.12 × 0.05
Diffractometer	Bruker APEX-II CCD	
Absorption correction	Multi-scan SADABS	
T_min_, T_max_	0.90, 0.98	0.92, 0.99
No. of measured, independent, and observed [I > 2σ(I)] reflections	37399, 2792, 2501	43034, 2369, 2703
R_int_	0.0292	0.0592
(sin θ/λ)_max_ (Å^–1^)	0.634	0.634
R[F^2^ > 2σ(F^2^)], wR(F^2^), S	0.0238, 0.0635, 1.051	0.0333, 0.0820, 1.032
No. of reflections	2792	3269
No. of parameters	171	220
H-atom treatment	H atoms treated by a mixture of independent and constrained refinement	
Δρ_max_, Δρ_min_ (e Å^–3^)	0.325, –0.206	0.297, –0.226

Computer programs: Bruker APEX3 [12], Bruker SAINT [12], SHELXL97 [13], ORTEP-3 for Windows [14], WinGX publication routines [15].

**Table 2:** Selected bond distances (Å).

Bond	ZZ1-04	ZZ1-13
C6–C7	1.4608 (17)	1.456 (2)
C7–N8	1.2817 (16)	1.284 (2)
N8–N9	1.3842 (14)	1.3835 (18)
N9–C10	1.3333 (16)	1.344 (2)
C10–S11	1.6726 (12)	1.6846 (16)
C10–S12/N12	1.7475 (13)	1.351 (2)

**Table 3:** Selected bond angles (°).

Bond	ZZ1-04	ZZ1-13
C6–C7–N8	121.93 (11)	123.08 (14)
C7–N8–N9	114.23 (10)	114.38 (13)
N8–N9–C10	120.75 (11)	121.15 (13)
N9–C10–S11	120.54 (9)	119.08 (12)
N9–C10–S12/N12	114.15 (9)	115.72 (14)
C10–S12/N12–C18	101.38 (6)	125.16 (14)
C3–O13–H13	107.2 (15)	109.1 (17)

**Table 4:** Parameters (Å, °) for hydrogen bonds and contacts in **ZZ1-04**.

D–H^…^A	D–H	H^…^A	D^…^A	D–H^…^A
O13–H13^…^S11 ^i^	0.76 (2)	2.66 (2)	3.3192 (10)	145.9 (18)
O13–H13^…^O15	0.76 (2)	2.226 (19)	2.6698 (13)	118.2 (17)
N9–H9^…^O13 ^ii^	0.859 (18)	2.108 (18)	3.0342 (14)	173.2 (16)

Symmetry codes: (i) x + 1, −y + 3/2, z + 1/2; (ii) x, −y + 3/2, z − 1/2.

**Table 5:** Parameters (Å, °) for hydrogen bonds and contacts in **ZZ1-13**.

D–H^…^A	D–H	H^…^A	D^…^A	D–H^…^A
N9–H9^…^S11 ^i^	0.83 (2)	2.64 (2)	3.4410 (14)	161.2 (18)
O13–O13^…^O14 ^ii^	0.81 (3)	2.35 (3)	2.9606 (16)	133 (2)
N12–H12^…^S11 ^iii^	0.83 (2)	2.78 (2)	3.4724 (14)	142.7 (17)

Symmetry codes: (i) −x + 2, −y + 3, −z; (ii) −x + 1, −y, −z; (iii) x, y − 1, z.

**Table 6:** SAP assay (assessed at A405 nm) comparing effects of old versus freshly prepared compounds. Values are the mean for n = 3 replicates.

Test Compound	A405 nm as % of Control
Only DMSO (1%) (Control Supernatant)	100%
Aged ZZ1-04	4.20%
Aged ZZ1-20	24.07%
Aged ZZ1-22	4.69%
Freshly Prepared ZZ1-04	9.35%
Freshly Prepared ZZ1-20	43.81%
Freshly Prepared ZZ1-22	114.47%

## Data Availability

The original contributions presented in the study are included in the article, further inquiries can be directed to the corresponding author.
